# Complete genome sequence of chicken anemia virus from a field outbreak in Bangladesh

**DOI:** 10.1128/mra.01095-24

**Published:** 2025-02-25

**Authors:** Marjana Akter, S. M. Nazmul Hasan, Nurejunnati Jeba, Anandha Mozumder, Moslema Jahan Mou, Yusha Araf, Roni Mia, Sharmin Akter, Md. Salauddin, Md Zaminur Rahman, Muhammad Shahdat Hossain, Sukumar Saha, Tofazzal Islam, Md. Golzar Hossain

**Affiliations:** 1 Department of Microbiology and Hygiene, Bangladesh Agricultural University 54492 , Mymensingh, Bangladesh; 2 Department of Physiology, Bangladesh Agricultural University 54492 , Mymensingh, Bangladesh; 3 Department of Microbiology and Public Health, Faculty of Veterinary, Animal and Biomedical Sciences, Khulna Agricultural University, Khulna, Bangladesh; 4 National Institute of Biotechnology, Savar, Dhaka, Bangladesh; 5 Institute of Biotechnology and Genetic Engineering, Bangabandhu Sheikh Mujibur Rahman Agricultural University 198780 , Gazipur, Bangladesh; Katholieke Universiteit Leuven, Leuven, Belgium

**Keywords:** chicken anemia virus, complete genome, Bangladesh

## Abstract

Chicken anemia virus (CAV) causes immunosuppression and increased mortality in chickens globally, including Bangladesh. This report provides the complete genome sequence of a CAV strain from a recent outbreak on a Bangladeshi layer farm, contributing to the limited availability of published data on the CAV genome in the country.

## ANNOUNCEMENT

Chicken anemia virus (CAV) is one of the most economically significant poultry viruses. The virus causes severe immunosuppression and anemia, which can lead to secondary bacterial infections. Mortality is often observed in flocks of 2–3-week-old chicks. CAV infection can interfere with immunization against other viruses, such as Newcastle disease virus (1). The virus is transmitted both horizontally and vertically, from infected hens to chicks. CAV is a non-enveloped virus about 25 nm in diameter with an icosahedral structure, classified under the *Anelloviridae* family, genus *Gyrovirus*, and species *Gyrovirus chicken anemia virus* (2). It contains a single-stranded DNA genome approximately 2298–2319 nucleotides in length (3). The genome encodes three overlapping viral genes: *vp1*, *vp2*, and *vp3* (4). Although the CAV has been circulating in many Asian countries, no complete genome sequence of Bangladeshi isolate has been reported over the last two decades.

A chicken from a layer farm in the Narsingdi District, Bangladesh (24.1344° N, 90.7860° E), suspected of having a CAV infection, was collected for analysis. The chickens of the farm exhibited signs of anemia and growth retardation. Postmortem analysis of the chicken revealed cyanosis, petechiae, ecchymoses, lethargy, pale comb, wattles, and eyelids. The femur of the chicken was cut lengthwise, and the bone marrow was gently flushed out with phosphate-buffered solution using a syringe into a 15 mL tube. The viral inoculum was prepared using our previously published protocol and stored at −80°C for further use (5). The viral DNA was extracted from the inoculum by TIANamp Virus DNA/RNA Kit (Tiangen, China) using the manufacturers' protocol. Next-generation sequencing of the extracted DNA was performed using the Illumina NovaSeq 6000. The entire library (Rapid Plus DNA Lib Prep Kit for Illumina, Cat# RK20208) was prepared through end repair, A-tailing, sequencing adapter addition, fragment screening, PCR amplification, and purification steps.

A total of 19,161,188 reads were generated after sequencing. Quality control was conducted using FastQC (v0.11.9). Raw reads were processed using Trimmomatic (v0.39) to trim low-quality bases and remove adapter sequences (6). Host DNA was removed using a computational pipeline from the sequencing reads before reference-guided sequence assembly, and then the reads were aligned with the chicken anemia virus reference genome (NCBI accession: OQ749509.1; genome size: 2432 bp) by using Burrows-Wheeler Aligner (BWA-men) (7) followed by viewing and sorting with Samtools (8), BCFtools (9), and VCFtools (10) and were then used to call the CAV consensus genome. Afterward, *de novo* genome assembly was carried out with the Unicycler tool for further verification (11). The genome was annotated using Prokka (v1.14.6) (12). The default parameters were used for all the abovementioned tools. The sequence coverage for this genome was 300×.

Unicycler successfully assembled the reads into a single contig of 2330 nucleotides, suggesting a complete assembly of the CAV genome (CAV/Narsingdi-1-MGH-BD), potentially covering the entire viral sequence. The closest relative/the reference genome was (NCBI accession: OQ749509.1; genome size: 2432 bp), and the genetic similarity/identity of this genome is 96%. The genome had a GC content of 42%. It comprised three major coding regions, which encode the VP1, VP2, and VP3 proteins. The lengths of the *vp1*, *vp2*, and *vp3* genes were 1350 bp (786 to 2135), 651 bp (313 to 963), and 366 bp (419 to 784), respectively. This report on the complete genome sequence of CAV from a recent outbreak in Bangladesh provides essential genomic information that could aid in developing vaccines to control and prevent infection in poultry.

## Data Availability

The complete genome sequence of the CAV isolate (CAV/Narsingdi-1-MGH-BD) has been deposited in GenBank, BioSample and SRA under accession numbers PQ412955, SAMN44002588, and PRJNA1167429, respectively.
